# Latent profile analysis of self-management and its association with quality of life differences in patients with cancer treated with immune checkpoint inhibitors

**DOI:** 10.1016/j.apjon.2025.100687

**Published:** 2025-03-13

**Authors:** Ruiqi Lu, Zhihui Yang, Jingxia Miao, Qian Xu, Lili Zhang

**Affiliations:** a School of Nursing, Southern Medical University, Guangzhou, China; b Department of Oncology, Nanfang Hospital, Guangzhou, China

**Keywords:** Immune checkpoint inhibitor, Latent profile analysis, Self-management, Quality of life, Immune-related adverse event

## Abstract

**Objective:**

This study aimed to explore latent profiles of self-management ability in patients with cancer treated with immune checkpoint inhibitors, analyze each subgroup's characteristics, and determine the relationship between self-management and quality of life.

**Methods:**

This cross-sectional study included 393 patients treated with immune checkpoint inhibitors. The participants completed questionnaires containing sociodemographic information, the Functional Assessment of Cancer Therapy-Immune Checkpoint Modulator (FACT-ICM), the Cancer Patient Self-management Evaluation Scale, and the Medical Coping Modes Questionnaire. Latent profile analysis was used to examine potential latent groups of self-management. Multivariate logistic regression was used to analyze the sociodemographic variables in each profile. Kruskal-Wallis H-rank sum test was used to explore the relationships between self-management profiles and quality of life.

**Results:**

The self-management abilities of the patients treated with immune checkpoint inhibitors were grouped into three latent profiles: “low self-management” (16.8%), “average self-management-avoidance of information” (44.3%), and “high self-management” (38.9%). The coping modes, educational levels, medical insurances, age, monthly family income per capita, and communication styles with health care professionals post-discharge significantly influenced the distribution of self-management. There were significant differences in the FACT-ICM scores across all three groups, except for the emotional well-being dimension.

**Conclusions:**

The patients with cancer treated with immune checkpoint inhibitors exhibit three distinct self-management profiles. To enhance patients' quality of life, healthcare professionals should develop targeted self-management strategies focusing on information management and communication between patients and healthcare providers.

## Introduction

Immunotherapies play a crucial role in cancer treatment, with immune checkpoint inhibitors (ICIs) being the most widely adopted immunotherapies due to their stable response and significant therapeutic effects in metastatic tumors.^1^ While ICIs exhibit anti-tumor effects through non-specific activation of the immune system, they can also cause several immune-related adverse events (irAEs).^2^ These irAEs occur in 66% or more and can involve multiple organs. Moreover, irAEs may occur at any time after initiation of immunotherapy and are a chief determinant of treatment prognosis and continuity of care in patients receiving ICIs.^3^ Most irAEs are reversible with early detection and appropriate treatment, without the need for hospitalization or interrupting immunotherapy.^4^ However, severe irAEs can lead to disruption of immunotherapy, increased healthcare costs, and even death.^5^ Medical and information needs related to irAEs are the two most critical concerns of patients treated with ICIs. Most patients and their caregivers are less informed about the manifestations, severity, and treatment of irAEs, thus increasing their reliance on healthcare professionals.^6^ Studies indicate that irAEs are highly prevalent in patients after discharge, yet the rate of active reporting of irAEs is low, and delays in reporting are common.^7^ The variable occurrence and duration of irAEs make it difficult for healthcare professionals to manage irAEs after discharge.

Self-management refers to a patient's ability to independently acquire knowledge and skills, manage treatment-related adverse events, and make emotional and social adjustments. Enhancement in self-management ability contributes to symptom relief and reduction in hospital days.^8^ Patients' self-management can facilitate the timely treatment of irAEs through intensive monitoring.^9^ Therefore, improving the self-management ability of patients treated with ICIs is a key determinant of disease prognosis. The multiple symptoms and stress caused by irAEs can reduce patients' quality of life (QoL).^10^ In this study, QoL was assessed using the Functional Assessment of Immune Checkpoint Modulators in Cancer Therapy Scale (FACT-ICM). The scale covers the assessment of multiple symptoms associated with irAEs and allows for a comprehensive assessment of QoL through emotional, social-family, and functional status.^11^ A study on adverse skin reactions to targeted therapy showed that patients with higher levels of self-management had better treatment adherence and reported a higher QoL.^12^ There is a correlation between QoL and patients' self-management ability. However, there is a lack of research on the self-management ability of patients with cancer treated with ICIs and its relationship with QoL.

Self-management of chronic diseases is mainly influenced by personal characteristics, health status, resources, and healthcare system factors.^13^ Personal characteristics include knowledge level and psychological distress. Age and educational levels are important determinants of patients' knowledge of irAEs.^14^ Coping styles reflect the cognitive and behavioral strategies adopted by patients in facing the pressures of the disease.^15^ Differences in coping styles have been found among patients with cancer have been associated with different levels of psychological resilience, highlighting the impact of coping styles on psychological well-being.^16^ Health status encompasses disease severity, course of treatment, and treatment side effects. Resources mainly refer to financial resources. Limited financial resources and lack of insurance reimbursement are the main barriers to self-management. Doctor–patient relationship is a key healthcare-related factor, and effective doctor-patient communication is essential to foster this relationship. Furthermore, coping styles play an important role in doctor-patient communication and information seeking.^17^

Previous studies have assessed total self-management ability scores separately from dimension-specific scores,^12^^,^^18^ but overlooked the existence of potential subgroups of self-management ability. Latent profile analysis (LPA) is a person-oriented approach based on individual response modeling, emphasizing the existence of heterogeneity.^19^ Identifying individual differences can provide statistical support for designing precise interventions.^20^ Based on levels of self-management, Xu et al.^21^ used LPA to classify lung cancer survivors into three heterogeneous subgroups: low self-management behavior-low emotion management group (11.90%), medium self-management behavior-low resource management group (45.24%), and high self-management behavior-low hope management group (42.86%). Such findings can help healthcare professionals explore weaknesses in self-management across groups and guide intervention to improve the self-management ability of patients in the corresponding subgroups.

The aims of this study were to (a) identify different latent profiles of self-management ability among patients treated with ICIs, (b) analyze the individual characteristics of each profile, and (c) determine the relationship between self-management and QoL.

## Methods

### *Study setting and sampling*

Convenience sampling was employed to recruit patients with cancer treated with ICIs who were hospitalized in the oncology department of a tertiary hospital in Guangzhou. Between January 2024 and July 2024, 393 eligible patients were invited to participate in the study. The inclusion criteria were as follows: age ≥18 years; pathologically diagnosed tumor; receipt of at least one cycle of ICI treatment; good communication and understanding of the questionnaire. Patients were excluded if they were in critical states that prevented participation in the study and their family members requested the use of protective medical measures for the patients. According to the rough estimation method of the sample size requirement of at least 300 cases for LPA,^19^ and considering 20% invalid questionnaires, the sample size was calculated to be at least 375 cases. This study met this sample size requirement.

### *Measures*

#### General information questionnaire

The investigators developed a custom sociodemographic questionnaire in consultation with clinical specialists and through a literature review. The questionnaire included information on age, gender, educational levels, occupational status, places of residence, time since diagnosis, types of tumor, cancer stages, types of ICIs, anti-tumor treatment regimens, cycles of immunotherapy completed, monthly family income per capita, types of immunotherapy, communication styles with healthcare professionals post-discharge, presence of irAEs, hospital admissions due to irAEs, and suspensions of ICIs due to irAEs.

#### Cancer patient self-management evaluation scale

Self-management ability was assessed using the self-management evaluation scale for patients with cancer developed by Chinese scholar Cheng in 2017.^22^ The scale encompasses six dimensions: daily activity management, symptom management, emotion management, communication with medical staff, information management, and self-efficacy. It consists of 44 items measured on a 5-point Likert scale from one (never) to five (always), with higher scores indicating better self-management ability. The Cronbach's *α* for the six dimensions ranged from 0.698 to 0.933, while the overall scale had a Cronbach's *α* of 0.889. The Cronbach's *α* of the scale in this study is 0.949.

#### Medical coping modes questionnaire

The Medical Coping Mode Questionnaire (MCMQ), originally developed by Feifel^15^ and translated into Chinese by Shen and Jiang in 2000,^23^ was used to assess patients' coping styles. The questionnaire consists of three dimensions and 20 items: the “facing” dimension (8 items), the “avoidance” dimension (7 items), and the “yield” dimension (5 items). The scale is scored on a four-point Likert scale from 1 (never) to 4 (always), with the three dimensions scored separately. Higher scores indicate a greater likelihood to use that coping style. The Cronbach's *α* coefficients for the three dimensions in this study are 0.888, 0.871, and 0.908.

#### Functional assessment of immune checkpoint modulators in cancer therapy scale

The FACT-ICM was employed to evaluate adverse events experienced by patients with cancer treated with ICIs and the impact of adverse events on their QoL. The scale was developed by Hansen et al.^11^ and later translated into Chinese by Meng.^24^ The scale consists of 52 items with five subscales: physical well-being, social/family well-being, emotional well-being, functional well-being, and the ICM subscale. Each item on the scale is scored on a five-point Likert scale from 0 (never) to 4 (always), with higher scores indicating better QoL. The overall retest reliability coefficient of the scale was 0.935, and in this study, the Cronbach's *α* for the scale is 0.926.

### *Data collection*

All researchers underwent standardized training to ensure that they used unified guidance to introduce the purpose, methods, and precautions of this study. The researchers provided eligible patients with detailed information about the purpose and voluntary nature of the study during hospitalization for treatments with ICIs. Those who agreed to sign an informed consent form were given a self-report questionnaire for data collection. All questionnaires were completed independently. For those who had difficulties completing the questionnaire by themselves, the researchers assisted in reading the questions and checking the answers without making any implications. After participants completed the survey, the researchers carefully checked all questionnaires to ensure completeness. The irAEs-related data were extracted from patients' medical records by researchers. Finally, 405 questionnaires were collected, of which 12 were excluded due to incomplete information, resulting 393 valid questionnaires and an effective recovery rate of 97.0%.

### *Data analysis*

Data analyses were performed using SPSS 25.0 and Mplus 8.3. LPA was conducted using Mplus 8.3 to determine the latent characteristics of self-management ability among patients receiving ICIs. The model fitting indices included the Akaike information criterion (AIC), Bayesian information criterion (BIC), and adjusted Bayesian information criterion (aBIC). Generally, lower AIC, BIC, and aBIC values are preferred for the LPA model. Larger entropy values indicate higher classification accuracy. An entropy greater than 0.80 indicates good classification accuracy.^25^ A significant Lo-Mendell-Rubin Likelihood Ratio Test (LMRT) probability (*P* < 0.05) indicate that the current model fits better than the previous model, while non-significant LMRT values (*P* > 0.05) indicate that the previous model is optimal.^26^

The Kolmogorov–Smirnov test was used to assess the normality of measurement data. Count data were described as frequency and percentage. Non-normally distributed measurement data were described using median (lower quartile, upper quartile). The chi-square test or Fisher's exact test was used to compare count data, while the Kruskal–Wallis H-rank sum test was used to compare non-normally distributed measurement data and ordinal data. Variables that showed significant differences (*P* < 0.05) in the univariate analysis were included in the multivariate logistic regression to further determine the factors affecting the distribution of subgroups. The Kruskal–Wallis H-rank sum test was used to compare the differences in QoL across profiles, and a post hoc test (Bonferroni test) was performed to further determine the differences in QoL between the categories. A two-tailed *P <* 0.05 was considered statistically significant.

## Results

Among the 393 respondents, 221 (56.2%) reported 14 types of irAEs. The most common irAE was skin toxicity (173 patients, 44.0%), followed by gastrointestinal toxicity (48, 12.2%), thyroid function toxicity (27, 6.9%), hepatotoxicity (18, 4.6%), pulmonary toxicity (7, 1.8%), and cardiotoxicity (6, 1.5%). Other toxicities were less frequent, including infusion reactions, eye toxicity, thrombocytopenia, arthritis, bladder toxicity, adrenal insufficiency, diabetes, and nephrotoxicity. Both the hospitalization rate and the treatment discontinuation rate of ICIs due to irAEs were 12.7%. Unplanned hospital admissions and discontinuation of ICIs were most commonly associated with hepatotoxicity (*n* = 12) among irAEs. The median age of the 393 patients treated with ICIs was 58.00 (51.00, 65.00) years. Additional patient characteristics are presented in Table 1. As shown in Table 1, age, medical coping modes, medical insurances, communication styles with healthcare professionals post-discharge, educational levels, and occupational status were statistically significant variables in the univariate analysis across different self-management profiles.

**Table 1:** Univariate analysis of respondents’ general information and potential categories of self-management (*N* = 393).

Variable	Total (*n* ​= ​393)	Profile 1 (*n* ​= ​66)	Profile 2 (*n* ​= ​174)	Profile 3 (*n* ​= ​153)	*χ*^*2*^*/H*	*P*
**Sex, *n* (%)**					5.171^a^	0.075
Male	280 (71.2)	43 (65.2)	134 (77.0)	103 (67.3)		
Female	113 (28.8)	23 (34.8)	40 (23.0)	50 (32.7)		
**Types of tumor, *n* (%)**					5.380^a^	0.496
Respiratory tumor^d^	235 (59.8)	41 (62.1)	109 (62.6)	85 (55.6)		
Digestive tumor^e^	100 (25.4)	16 (24.2)	41 (23.6)	43 (28.1)		
Head and Neck tumor^f^	30 (7.6)	2 (3.0)	14 (8.0)	14 (9.2)		
Other tumor^g^	28 (7.1)	7 (10.6)	10 (5.7)	11 (7.2)		
**Educational levels, *n* (%)**					23.858^b^	< 0.001
Primary school or lower	114 (29.0)	31 (47.0)	62 (35.6)	21 (13.7)		
Middle school	117 (29.8)	21 (31.8)	61 (35.1)	35 (22.9)		
Senior high school	101 (25.7)	10 (15.2)	36 (20.7)	55 (35.9)		
College/university or higher	61 (15.5)	4 (6.1)	15 (8.6)	42 (27.5)		
**Time since diagnosis, *n* (%)**					1.738^b^	0.419
< 6 months	125 (31.8)	27 (40.9)	58 (33.3)	40 (26.1)		
6–12 months	131 (33.3)	17 (25.8)	54 (31.0)	60 (39.2)		
> 12 months	137 (34.9)	22 (33.3)	62 (35.6)	53 (34.6)		
**Cancer stage, *n* (%)**					2.150^b^	0.341
I/II	18 (4.6)	0 (0.0)	9 (5.2)	9 (5.9)		
III	65 (16.5)	10 (15.2)	29 (16.7)	26 (17.0)		
IV	310 (78.9)	56 (84.8)	136 (78.2)	118 (77.1)		
**Cycles of immunotherapy completed, *n* (%)**					1.077^a^	0.584
≤ 6 cycles	228 (58.0)	42 (63.6)	98 (56.3)	88 (57.5)		
> 6 cycles	165 (42.0)	24 (36.4)	76 (43.7)	65 (42.5)		
**Anti-tumor treatment regimens, *n* (%)**					9.595^a^	0.143
Immunotherapy	59 (15.0)	3 (4.5)	26 (14.9)	30 (19.6)		
Immunotherapy ​+ ​Chemotherapy	183 (46.6)	33 (50.0)	84 (48.3)	66 (43.1)		
Immunotherapy ​+ ​Targeted therapy	72 (18.3)	14 (21.2)	28 (16.1)	30 (19.6)		
Immunotherapy ​+ ​Chemotherapy ​+ ​Targeted therapy	79 (20.1)	16 (24.2)	36 (20.7)	27 (17.6)		
**Type of ICIs, *n* (%)**						
PD-1 Inhibitors^h^	370 (94.1)	63 (95.5)	165 (94.8)	142 (92.8)	–c	0.811
PD-L1 Inhibitors^i^	20 (5.1)	3 (4.5)	7 (4.0)	10 (6.5)		
PD-1 and CTLA-4 bispecific antibody^j^	3 (0.8)	0 (0.0)	2 (1.1)	1 (0.7)		
**Occupational status, *n* (%)**						
Employment	82 (20.9)	2 (3.0)	14 (8.0)	66 (43.1)	84.385^a^	<0.001
Medical leaves	67 (17.0)	14 (21.2)	33 (19.0)	20 (13.1)		
Retirement	139 (35.4)	21 (31.8)	72 (41.4)	46 (30.1)		
Unemployment	105 (26.7)	29 (43.9)	55 (31.6)	21 (13.7)		
**Medical insurances, *n* (%)**					15.524^a^	0.004
Urban employee insurance/ commercial insurance	158 (40.2)	15 (22.7)	73 (42.0)	70 (45.8)		
Urban residents insurance	117 (29.8)	20 (30.3)	49 (28.2)	48 (31.4)		
None or rural cooperative insurance	118 (30.0)	31 (47.0)	52 (29.9)	35 (22.9)		
**Residence, *n* (%)**					2.092^a^	0.719
Urban	147 (37.4)	29 (43.9)	65 (37.4)	53 (34.6)		
Town	128 (32.6)	18 (27.3)	59 (33.9)	51 (33.3)		
Rural	118 (30.0)	19 (28.8)	50 (28.7)	49 (32.0)		
**Communication styles with healthcare professionals post-discharge, *n* (%)**					42.626^a^	< 0.001
Family members communicate on behalf of the patient	151 (38.4)	34 (51.5)	77 (44.3)	40 (26.1)		
Communicate directly by themselves	135 (34.4)	14 (21.2)	39 (22.4)	82 (53.6)		
Lack of active communication	107 (27.2)	18 (27.3)	58 (33.3)	31 (20.3)		
**Monthly family income per capita (¥), *n* (%)**					50.934^b^	< 0.001
> 8000	65 (16.5)	2 (3.0)	20 (11.5)	43 (28.1)		
5000–7999	98 (24.9)	7 (10.6)	42 (24.1)	49 (32.0)		
2000–4999	129 (32.8)	29 (43.9)	59 (33.9)	41 (26.8)		
< 2000	101 (25.7)	28 (42.4)	53 (30.5)	20 (13.1)		
**Presence of irAEs, *n* (%)**					0.077^a^	0.962
No irAEs	172 (43.8)	28 (42.4)	76 (43.7)	68 (44.4)		
Occur irAEs	221 (56.2)	38 (57.6)	98 (56.3)	85 (55.6)		
**Hospital admissions due to irAEs, *n* (%)**						
No	343 (87.3)	56 (84.8)	146 (83.9)	141 (92.2)	5.410^a^	0.067
Yes	50 (12.7)	10 (15.2)	28 (16.1)	12 (7.8)		
**Suspensions of ICIs due to irAEs, *n* (%)**					5.824^a^	0.054
No	343 (87.3)	54 (81.8)	148 (85.1)	141 (92.2)		
Yes	50 (12.7)	12 (18.2)	26 (14.9)	12 (7.8)		
Age (Years), *M(P25, P75)*	58.00 (51.00, 65.00)	59.50 (51.00, 67.00)	61.00 (53.75, 66.00)	55.00 (49.00, 65.00)	21.499^b^	< 0.001
**Medical coping modes, *M(P25,P75)***						
Facing	19.00 (15.00, 23.00)	13.50 (10.00, 18.00)	17.00 (14.75, 20.00)	24.00 (21.00, 27.00)	188.467^b^	< 0.001
Avoidance	20.00 (16.00, 24.00)	12.50 (10.00, 16.00)	19.00 (17.00, 22.00)	23.00 (21.00, 26.00)	170.864^b^	< 0.001
Yield	8.00 (6.00, 11.00)	15.50 (12.75, 18.00)	8.00 (7.00, 10.00)	6.00 (5.00, 7.00)	189.936^b^	< 0.001

a *χ^2^*-value.

b *H*-value.

c Fisher's exact test.

d Indicates to lung cancer and mediastinal tumors.

e Indicates to esophageal cancer, gastric cancer, liver cancer and colorectal cancer.

f Indicates to nasopharyngeal cancer, oral cancer, parotid tumours and thyroid cancer.

g Indicates to bladder cancer, kidney cancer, uterine cancer, melanoma and osteosarcoma.

h Indicates to programmed cell death protein 1 (PD-1).

i Indicates to programmed cell death ligand 1 (PD-L1).

j Indicates to Candonilimab, the PD-1/CTLA-4 (cytotoxic T-lymphocyte-associated protein 4) bispecific antibody.

### *Latent profile determination*

A total of four models were fitted in this study, as shown in Table 2. The AIC, BIC, and aBIC indices decreased as the number of categories increased. The information entropy reached the highest value (0.936) when the number of profiles was three but decreased when the number of profiles was four. Considering the fit indices and the practical significance of classification, this study selected models with three profiles as the best latent profile model for self-management of patients with cancer treated with ICIs. The probabilities of patients being classified to each profile were 0.992, 0.978, and 0.973.

**Table 2:** Fit indices of latent profile of self-management (*N* ​= ​393).

Model	AIC	BIC	aBIC	Entropy	LMRT (*P*)	BLRT (*P*)	Probability of profile
1	4974.216	5021.902	4983.826	–	–	–	–
2	4117.400	4192.902	4132.616	0.935	0.005	< 0.001	0.198/0.802
3	3619.990	3723.309	3640.811	0.936	< 0.001	< 0.001	0.168/0.443/0.389
4	3532.544	3663.679	3558.971	0.934	0.043	< 0.001	0.054/0.122/0.435/0.389

AIC, ​Akaike Information Criterion; BIC, Bayesian Information Criterion; aBIC, ​Adjusted Bayesian Information Criterion; LMRT, ​Lo-Mendell-Rubin likelihood ratio test; BLRT, Bootstrapped likelihood ratio test.

**Fig. 1:**
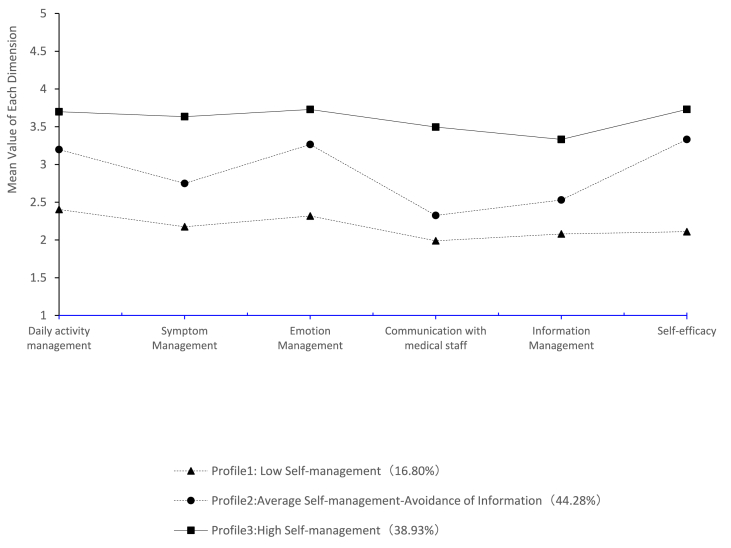
Latent profiles of self-management in patients treated with ICIs. ICIs, immune checkpoint inhibitors. (Due to the different number of items in each dimension of self-management, the vertical scale scores are depicted using the mean of scores for each dimension, calculated as the total score for each dimension divided by the number of items in that dimension)

Fig. 1 illustrates the mean scores of self-management profiles. Profile 1 represented 16.8% of the sample (*n* = 66) and had a total self-management score of 100.50 (91.50, 112.00). This group had the lowest level across all dimensions and was therefore classified as the “Low Self-management Group.” Profile 2 represented 44.3% of the sample (*n* = 174). This subgroup had an average self-management score of 135.00 (127.00, 141.00) and lower mean scores on communication with healthcare professionals and information communication. Thus, this profile was labeled the “Average Self-management-Avoidance of Information Group.” Profile 3 included 153 cases (38.9%) and had the highest self-management score at 161.00 (155.00, 166.50). Therefore, the subgroup was designated as the “High Self-management Group.”

### *Multifactor analysis of potential profiles of self-management in patients treated with ICIs*

As shown in Table 3, we conducted multivariate logistic regression analyses with the three latent profiles as the dependent variable, using ‘low self-management’ as the reference group, and six significant variables from the univariate analyses as the independent variables. The model explained 73.0% of the variance across the three profiles of self-management.

**Table 3:** Multiple logistic regression of sociodemographic variables on subgroups of self-management (*N* = 393).

Variables	Profile 2					Profile 3				
	*β*	S.E	*P*	OR	95% CI	*β*	S.E	*P*	OR	95% CI
Age	0.128	0.041	0.002	1.136	1.048–1.232	0.088	0.048	0.066	1.092	0.994–1.199
Facing	0.267	0.081	0.001	1.306	1.114–1.530	0.793	0.110	< 0.001	2.211	1.782–2.743
Avoidance	0.253	0.099	0.010	1.288	1.062–1.562	0.409	0.117	< 0.001	1.506	1.198–1.892
Yield	−0.490	0.111	< 0.001	0.612	0.493–0.761	−0.800	0.153	< 0.001	0.449	0.333–0.606
**Medical insurance (Ref: None or rural cooperative insurance)**										
Urban employee insurance/commercial insurance	1.512	0.667	0.023	4.537	1.227–16.779	2.112	0.807	0.009	8.268	1.699–40.234
Urban residents insurance	−0.100	0.702	0.887	0.905	0.229–3.580	−0.319	0.864	0.712	0.727	0.134–3.954
**Communication styles with healthcare professionals post-discharge (Ref: Lack of active communication)**										
Family members communicate on behalf of the patient	−1.086	0.694	0.117	0.337	0.087–1.315	0.018	0.862	0.983	1.018	0.188–5.518
Communicate directly by themselves	−0.322	0.757	0.671	0.725	0.164–3.198	2.325	0.935	0.013	10.230	1.636–63.964
**Educational levels (Ref: Primary school or lower)**										
Middle school	1.037	0.649	0.110	2.822	0.791–10.073	1.476	0.834	0.077	4.375	0.854–22.416
Senior high school/Vocational school	1.104	0.808	0.172	3.015	0.619–14.682	1.968	0.975	0.044	7.158	1.059–48.378
College/University or higher	2.631	1.301	0.043	13.893^a^	1.086–177.796^a^	2.871	1.447	0.047	17.662^a^	1.037–300.859^a^
**Occupational status (Ref: Unemployment)**										
Employment	1.692	1.545	0.273	5.430	0.263–112.132	2.824	1.652	0.087	16.848	0.661–429.633
Medical leaves	−0.817	0.755	0.280	0.442	0.101–1.942	−0.984	0.924	0.287	0.374	0.061–2.285
Retirement	−0.350	0.759	0.645	0.705	0.159–3.120	0.953	0.995	0.338	2.593	0.369–18.241
**Monthly family income per capita (¥,Ref: <2000)**										
> 8000	0.255	2.629	0.923	1.290	0.007–222.932	0.325	2.703	0.904	1.384	0.007–276.435
5000–7999	−0.302	0.806	0.708	0.739	0.152–3.586	−0.604	0.984	0.540	0.547	0.079–3.763
2000–4999	−1.621	0.692	0.019	0.198	0.051–0.767	−2.094	0.846	0.013	0.123	0.023–0.646

CI, confidence interval; OR, odds ratio; Ref, reference.

a Indicates that the OR is unusually large and the CI is unusually wide, which indicates the variable has little frequency under a classification.

Facing (average self-management-avoidance of information: odds ratio [OR] = 1.306, 95% confidence interval [CI] [1.114, 1.530]; high self-management: OR = 2.211, 95% CI [1.782, 2.743]), avoidance (average self-management-avoidance of information: OR = 1.288, 95% CI [1.062, 1.562]; high self-management: OR = 1.506, 95% CI [1.198, 1.892]), yield (average self-management-avoidance of information: OR = 0.612, 95% CI [0.493, 0.761]; high self-management: OR = 0.449, 95% CI [0.333, 0.606]), having a university degree or higher (average self-management-avoidance of information: OR = 13.893, 95% CI [1.086, 177.796]; high self-management: OR = 17.662, 95% CI [1.037, 300.859), and purchasing urban employee insurance or commercial insurance (‘average self-management-avoidance of information profile’: OR = 4.537, 95% CI [1.227, 16.779]; ‘high self-management’: OR = 8.268, 95% CI [1.699, 40.234]) were factors influencing latent profiles. Patients who preferred to communicate directly by themselves (OR = 10.230, 95% CI [1.636, 63.964]) were more likely to belong to the ‘high self-management’ than the ‘low self-management’ group. Older patients (OR = 1.136, 95% CI [1.048, 1.232]) were more likely to belong to the ‘average self-management-avoidance of information’ group. Patients with a monthly family income per capita of ¥2000–4999 were less likely to be classified into the ‘average self-management-avoidance of information’ group (OR = 0.198, 95% CI [0.051, 0.767]) or ‘high self-management’ group (OR = 0.123, 95% CI [0.023, 0.646]).

### *Relationships between the latent profiles of self-management and QoL*

**Fig. 2:**
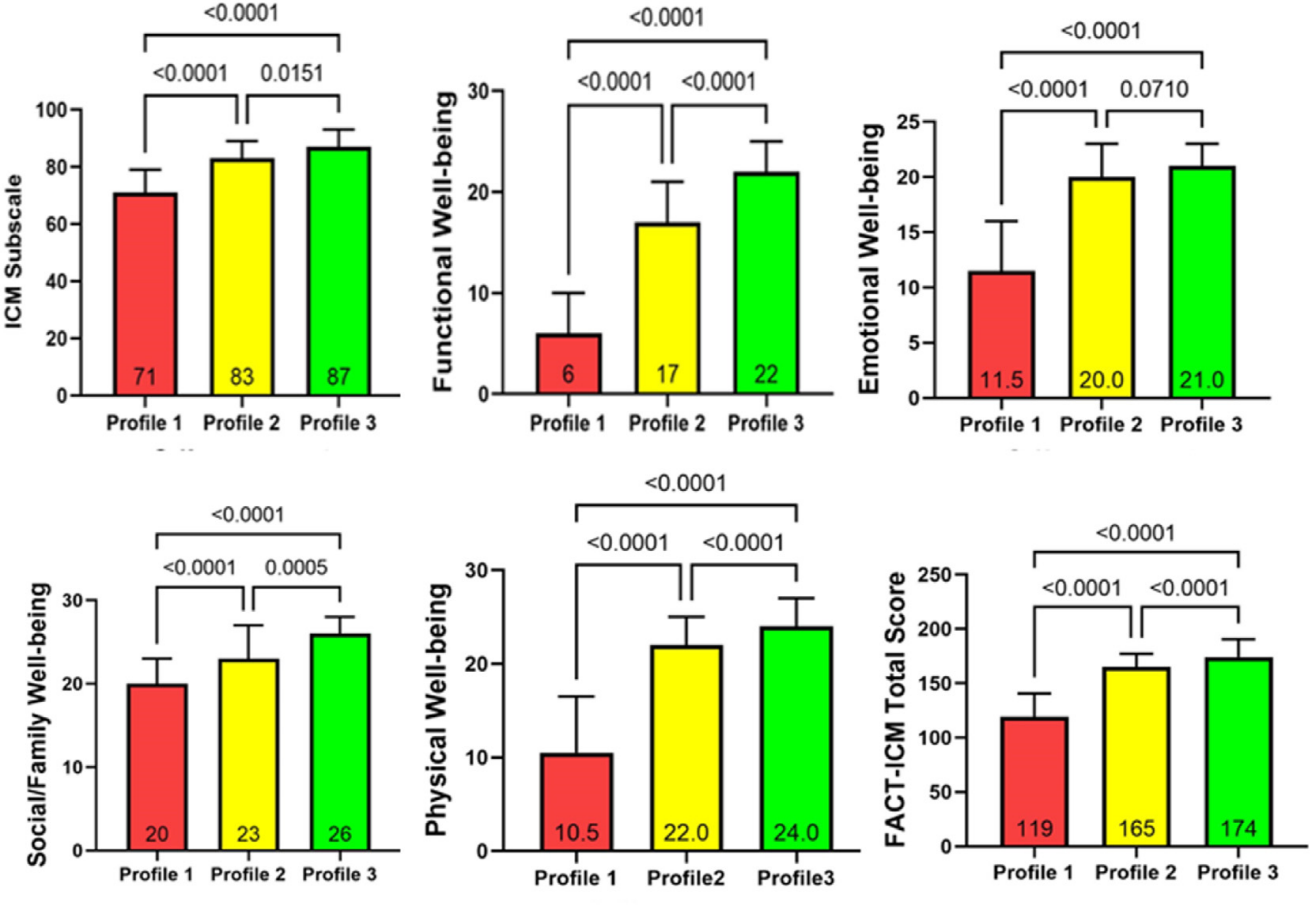
Comparison of dimensions and total scores of FACT-ICM among patients treated with ICIs in different self-management profiles. FACT-ICM, Functional Assessment of Cancer Therapy-Immune Checkpoint Modulator; ICIs, immune checkpoint inhibitors. (Profile 1 ​= ​Low Self-management Profile, Profile 2 ​= ​Average Self-management-Avoidance of Information Profile, Profile 3 ​= ​High Self-management Profile).

The total FACT-ICM score in this study was 164.00 (140.50, 180.00). As shown in Fig. 2, the three subgroups were statistically different on all six dimensions of the FACT-ICM scale (*P* < 0.01). In the dimension of emotional well-being, there was no significant difference between the ‘average self-management-avoidance of information’ group and the ‘high self-management’ group (*P* = 0.071). For all other dimensions and the total score, the ‘high self-management’ group had the highest scores, followed by the ‘average self-management-avoidance of information’ group, while the ‘low self-management’ group had the lowest scores.

## Discussion

The incidence of irAEs in this study was 56.2%, which is slightly higher than the incidence of irAEs reported in the previous studies.^27^^,^^28^ However, there was no significant difference in the incidence of irAEs across different self-management profiles. The rate of hospitalization due to irAEs was similar to the rate observed in a previous study.^27^ Previous studies have shown that the most common irAEs requiring hospital admission are colitis, hepatotoxicity, and pulmonary toxicity.^29^ Hepatitis was the most frequent cause of hospital admissions in this study, which may be due to the later onset of colitis and pulmonary toxicity.^28^^,^^30^ Additionally, most of the patients in this study received fewer cycles of ICIs treatments. High-grade irAEs usually resolve after withholding ICIs and systemic use of immunosuppressant agents. Patients who restart ICIs after experiencing severe irAEs have a higher incidence of irAEs compared to those receiving ICIs for the first time.^31^ The interval between the occurrence and treatment of irAEs affects prognosis.^10^ Severe irAEs increase the burden of treatment and therefore require prompt management to prevent exacerbation of irAEs.

This study is the first to use LPA to explore the characteristics of self-management ability in patients receiving ICIs. The study demonstrated a high self-management score of 141 (126, 157) compared to a score of 135 (105, 157) in another study on the self-management ability of patients with lung cancer experiencing adverse skin reactions to targeted therapies.^12^ Through LPA, this study classified three subgroups of self-management of patients with cancer treated with ICIs.

Results showed that 16.8% of the patients belonged to the low self-management group, characterized by low scores on all dimensions. This suggests that patients in this group were less motivated to manage their disease and had limited knowledge of the disease and treatment-related adverse events. Additionally, this group of patients tended to adopt the yield coping style, perceiving loss of control of their health and difficulty in dealing with the stressful changes associated with treatment with ICIs. For this group of patients, healthcare professionals should encourage patients to confront their illnesses and adverse events, provide accessible education on irAEs, and develop feasible patient self-management measures based on their specific conditions. Consistent with findings in patients with lung cancer receiving ICIs, those with lower income were more likely to be categorized in the low self-management group.^32^ These patients may find it difficult to afford the cost of ICIs treatment and daily expenses, creating significant financial pressure that will diminish the motivation to take control of their health condition.^13^

A larger proportion of patients (44.3%) were classified into the average self-management-avoidance of information group. Patients in this group scored higher than those with low self-management in all areas, with self-efficacy being the area in which they scored highest, followed by emotion management. Similar to the low self-management group, this group scored the lowest on the dimension of communication with medical staff, followed by information management. This reflects that patients in this group are more confident in managing their health conditions and willing to resolve their negative emotions by expressing feelings and distractions. However, they were less willing to engage in illness consultation as well as medical decision-making and were less likely to acquire health-related knowledge through other means. Older patients were more likely to be categorized in this group, which may be attributed to the limited perception of irAEs in older people and the greater emotional stability of older people since they are more likely to accept the present state of the disease.^33^^,^^34^ Nevertheless, older patients often face challenges in accessing and recognizing reliable health information.^35^ For these patients, healthcare professionals should implement more ways to communicate with them and encourage them to share their disease management experiences with each other.

The high self-management group contained 38.9% of the patients. This group was characterized by highest scores across all dimensions, with slightly lower scores for information management. This indicates that this group still had limited ability to effectively acquire information related to ICIs, which may be due to the low popularization of knowledge related to ICIs.^36^ Healthcare providers should enrich the format of health education materials related to ICIs and irAEs, improve their readability and accessibility, and evaluate the effectiveness of the delivery.

The high self-management group shared some influencing factors with the average self-management group. For example, patients in the two groups tended to use facing and avoidance coping styles. The results are consistent with the previous findings.^16^ Patients with high facing scores were able to actively acquire knowledge and actively engage in communication. Patients with higher avoidance scores tended to divert their attention from the disease, and therefore, their daily lives were less likely to be disrupted. Additionally, patients covered by urban health insurance and commercial insurance were more likely to fall into these two categories. These health insurance plans cover a large portion of medical expenses and provide financial security for self-management.^37^ Similar to the results of previous studies,^38^ patients with cancer who have higher levels of education were more likely to be in these two groups. This may be because more educated patients understand the mechanism of action of ICIs and the uncertainty of the occurrence of irAEs compared to less educated patients.^14^

It is noteworthy that patients with high self-management ability preferred to communicate directly with healthcare professionals by themselves. Delay in seeking help for irAEs prevents timely management, especially if the patient perceives symptoms of irAEs as normal reactions to treatment.^39^ However, patients have difficulty determining the cause of the symptoms and prefer to wait until their next appointment to discuss symptoms that occurred after discharge with their clinicians. Studies have shown that the main reasons for patients do not report ICI-related symptoms include their belief that the symptoms are not severe enough and fear of stopping treatment. In contrast, the desire to know if symptoms are normal and the willingness to monitor response to treatment prompts patients to report symptoms.^40^ Patients' behaviour of reporting symptoms is an important aspect of self-management. Developing risk awareness can help patients to understand the link between symptoms and irAEs, and to report them promptly to their physicians for differential diagnosis, reducing readmission rates due to irAEs.^41^^,^^42^ Therefore, patients should be encouraged to participate in identifying and reporting irAEs.

The total FACT-ICM score in the study was lower than the score of 171.00 (159.75,184.00) reported by a previous study of patients with Karnofsky performance status ≤ 80.^24^ Patients with higher self-management ability have a higher QoL, which is consistent with previous findings.^18^ The application of self-management strategies helps patients cope with symptoms and maintain a positive mindset so as to reduce disease burden and improve the QoL.

### *Implications for nursing practice and research*

The self-management ability of patients with cancer treated with ICIs can be categorized as “low,” “average,” and “high.” For the low self-management group, healthcare professionals should take the initiative to teaching patients to cope with their symptoms and to make appropriate emotional adjustments. For the average group, healthcare professionals should mobilize patients to participate in doctor-patient communication, organize group learning, and encourage patients to share their experiences of irAEs management. The high self-management group has space for improvement in information management, so healthcare professionals are required to provide patients with more access to knowledge about ICIs and irAEs.

This study identified relevant factors affecting the distribution of self-management, including coping styles, age, educational levels, health insurances, monthly family income per capita, and communication styles with healthcare professionals after discharge. Identifying relevant factors that influence self-management can assist healthcare providers in tailoring interventions to improve the self-management ability of patients. Healthcare professionals should encourage patients to confront the disease positively and divert their attention appropriately to avoid yielding to the disease. Nurses should provide health education that meets the cognitive level of elderly patients and patients with low educational attainment. For patients with low income or who don't have urban employee health insurance or commercial insurance, nurses should communicate with patients about financial issues and discuss the resources available to them to reduce the financial burden of disease management. Nurses should encourage patients treated with ICIs to proactively engage in symptom reporting for early identification and management of irAEs. This study suggests that patients with high levels of self-management have a better QoL, indicating that to improve the QoL of patients treated with ICIs, healthcare professionals should prioritize improving patients' self-management skills along with regular diagnosis and treatment.

Longitudinal studies are needed to investigate the trajectory of self-management ability throughout the course of immunotherapy. Additionally, healthcare professionals should enhance follow-up to accurately assess the time to onset and severity of irAEs and to assist patients in self-management.

### *Limitations*

This study collected data from a tertiary hospital in a single province in China, which may have geographical limitations. Due to the imbalance in the number of cancer types among patients admitted to the hospital, the proportion of cancer types in each self-management subgroup was not consistent, which may lead to non-significant differences in the distribution of self-management subgroups across cancer types. The effect of cancer types on self-management requires further verification. Findings from this cross-sectional study could not accurately reflect the trends in irAEs, QoL, or self-management ability across ICIs treatment cycles. This study did not report on the severity of irAEs because irAEs often occur after discharge. Most patients received irAEs-related diagnoses and treatments in nearby facilities, making it difficult to obtain medical records at that time of occurrence of irAEs for adequate assessment. The investigators could not assess the severity of irAEs in each case due to recall bias and inadequate medical data.

## Conclusions

This study used LPA to identify latent profiles of self-management ability of patients with cancer treated with ICIs. Information management and communication with healthcare providers were the two lowest-scoring dimensions, highlighting potential weaknesses in self-management. Patients' self-management ability can be categorized into three subgroups, which were significantly correlated with coping modes, educational levels, medical insurances, age, monthly family income per capita, and communication styles with healthcare professionals post-discharge. Patients with higher self-management ability tend to have better QoL among patients treated with ICIs. Healthcare professionals should identify self-management characteristics and implement tailored self-management enhancement strategies focusing on information management and communication between patients and healthcare providers to improve the QoL of patients treated with ICIs.

## CRediT authorship contribution statement

**Ruiqi Lu**: Conceptualization, Data curation, Formal analysis, Investigation, Visualization, Writing - original draft. **Zhihui Yang**: Conceptualization, Funding acquisition, Formal analysis, Methodology, Project administration, Writing - review & editing. **Jingxia Miao**: Resources, Project administration, Supervision. **Qian Xu**: Resources, Investigation. **Lili Zhang**: Conceptualization, Project administration, Supervision, Writing - review & editing. All authors had full access to all the data in the study, and the corresponding author had final responsibility for the decision to submit for publication. The corresponding author attests that all listed authors meet authorship criteria and that no others meeting the criteria have been omitted.

## Ethics statement

The study was conducted in accordance with the principles stated in the Declaration of Helsinki and was approved by the Medical Ethics Committee of Nanfang Hospital of Southern Medical University (IRB No. NFEC-2022-080). All participants provided written informed consent.

## Data availability statement

The data that support the findings of this study are available from the corresponding author, Lili Zhang, upon reasonable request.

## Declaration of generative AI and AI-assisted technologies in the writing process

No AI tools/services were used during the preparation of this work.

## Funding

This study was supported by the Youth Project of the 10.13039/501100001809National Natural Science Foundation of China (Grant No. 72204106). The funders had no role in considering the study design or in the collection, analysis, interpretation of data, writing of the report, or decision to submit the article for publication.

## Declaration of competing interest

The authors declare no conflicts of interest.
